# Context-Dependent Modulation of Ferroptosis by Metformin: Mechanisms, Therapeutic Implications and Open Questions

**DOI:** 10.3390/ph19071072

**Published:** 2026-07-11

**Authors:** Nail Besli, Nilufer Ercin, Rabia Kalkan Cakmak, Ulkan Celik

**Affiliations:** 1 Department of Medical Biology, Hamidiye School of Medicine, University of Health Sciences, Istanbul 34668, Türkiye; 2 Department of Medical Physiology, Graduate School, Istanbul Medeniyet University, Istanbul 34700, Türkiye; 3 Department of Medical Biology, Faculty of Medicine, Istanbul Atlas University, Istanbul 34408, Türkiye

**Keywords:** ferroptosis, metformin, AMPK, lipid peroxidation, iron metabolism, cancer therapy, neuroprotection

## Abstract

Ferroptosis is an iron-dependent regulated form of cell death characterized by lethal lipid peroxidation and is increasingly implicated in cancer, neurodegenerative diseases, cardiovascular injury, and metabolic disorders. Metformin, a widely prescribed antidiabetic biguanide, exerts pleiotropic effects beyond glucose lowering and has emerged as a context-dependent regulator of ferroptosis. In malignant cells, metformin may enhance ferroptotic susceptibility through activation of AMP-activated protein kinase (AMPK), suppression of mechanistic target of rapamycin (mTOR) signaling and SLC7A11, induction of ferritinophagy, mitochondrial complex I stress, and promotion of lipid peroxidation. Conversely, in normal or stressed non-malignant tissues, metformin may limit ferroptotic injury by activating nuclear factor erythroid 2-related factor 2 (NRF2), supporting glutathione peroxidase 4 (GPX4) and SLC7A11-dependent antioxidant defenses, improving mitochondrial quality control, and stabilizing iron homeostasis. This review synthesizes the molecular basis of this duality, evaluates therapeutic opportunities in oncology and cytoprotection, and outlines biomarker-driven and clinical trial strategies required for translation. Overall, metformin should not be regarded as a universal ferroptosis inducer or inhibitor, but rather as a context-dependent metabolic regulator whose effects are shaped by cell type, dose, exposure duration, transporter expression, iron status, and antioxidant capacity.

## 1. Introduction

Ferroptosis represents a regulated form of cell death that is mechanistically distinct from apoptosis, necroptosis, and accidental necrosis. Its hallmark biochemical feature is the iron-dependent accumulation of lethal lipid peroxides within cellular membranes, especially in phospholipids containing polyunsaturated fatty acyl (PUFA) chains. Since its initial characterization as an iron-dependent, non-apoptotic cell death modality, ferroptosis has emerged as a central concept connecting redox biology, lipid metabolism, iron homeostasis, and disease pathogenesis [1,2,3].

The execution and suppression of ferroptosis are controlled by several interconnected molecular modules. The system Xc^−^-GSH-GPX4 axis supplies cystine for glutathione synthesis and enables GPX4 to detoxify lipid hydroperoxides into non-toxic lipid alcohols [4]. Iron metabolism determines the size and reactivity of the labile iron pool, thereby influencing Fenton chemistry and radical generation [5]. Lipid metabolic enzymes, particularly ACSL4 and LPCAT3, regulate the incorporation of PUFA-containing phospholipids into membranes and therefore determine the availability of substrates vulnerable to peroxidation [6]. In parallel, GPX4-independent defense systems, including FSP1-CoQ10, DHODH-CoQ10, GCH1-BH4, vitamin K cycling, and the 7-dehydrocholesterol pathway, provide additional layers of resistance against lipid peroxidation [7,8,9,10].

Metformin, a biguanide and first-line antidiabetic drug, exhibits biological effects that extend beyond glucose lowering. It modulates mitochondrial metabolism, AMPK activity, inflammatory signaling, cancer cell metabolism, cardiovascular outcomes, and age-related processes [11,12]. Although metformin is traditionally associated with AMPK activation and inhibition of mitochondrial respiratory-chain complex I, its downstream effects vary considerably depending on cellular energetic state, tissue distribution, transporter expression, dose, and exposure duration [13]. This context dependency is particularly relevant to ferroptosis, as identical upstream metabolic stressors can yield opposite biological outcomes depending on whether a cell is near an oxidative threshold or possesses sufficient adaptive antioxidant reserves [14].

An increasing body of experimental evidence indicates that metformin can either promote or suppress ferroptosis, depending on the context of the disease. In malignant cells, characterized by high iron demand, elevated reactive oxygen species, and reliance on antioxidant buffering, metformin may promote ferroptotic cell death by modulating AMPK-mTOR-SLC7A11 signaling, mitochondrial stress, ferritinophagy, and lipid peroxidation. In contrast, in non-malignant or stressed tissues, metformin may facilitate adaptive cytoprotection by enhancing NRF2-dependent antioxidant responses, mitochondrial quality control, and iron homeostasis. Despite these divergent effects, discussions often remain compartmentalized within cancer, cardiovascular, neurological, or metabolic disease literature, resulting in a significant conceptual gap [15,16,17].

This review addresses the absence of an integrated framework that explains why metformin acts as a pro-ferroptotic sensitizer in certain contexts and as an anti-ferroptotic cytoprotective agent in others. Rather than categorizing metformin as a universal ferroptosis inducer or inhibitor, the review proposes that metformin functions as a context-dependent metabolic rheostat. The molecular mechanisms underlying this duality are synthesized, and the influence of disease state, dose, exposure duration, transporter expression, iron status, and antioxidant capacity on ferroptotic outcomes is evaluated. Additionally, the review discusses the necessity of biomarker-guided strategies before metformin can be rationally implemented as a ferroptosis-directed therapeutic intervention.

## 2. Molecular Mechanisms of Metformin-Mediated Ferroptosis Modulation

Metformin appears to regulate ferroptosis through multiple interconnected biochemical pathways rather than via a single linear mechanism. AMPK activation induced by metformin can suppress mTOR signaling, thereby reshaping cellular metabolic and redox homeostasis and modifying the requirement for antioxidant systems such as glutathione. AMPK-mTOR crosstalk also intersects with cystine import mediated by system Xc^−^, while simultaneously influencing mitochondrial function, iron metabolism, and lipid peroxidation. Consequently, the net effect of metformin on ferroptotic susceptibility is likely determined by the dynamic balance among these regulatory axes. Such balance may differ according to cell type, metabolic context, and the availability of compensatory antioxidant defenses. Growing evidence further suggests that ferroptosis contributes to aging and neurodegenerative disorders, in which mTOR-driven anabolic activity may sustain elevated antioxidant demand and increase reliance on system Xc^−^-dependent cystine uptake [15,18,19].

**Figure 1:**
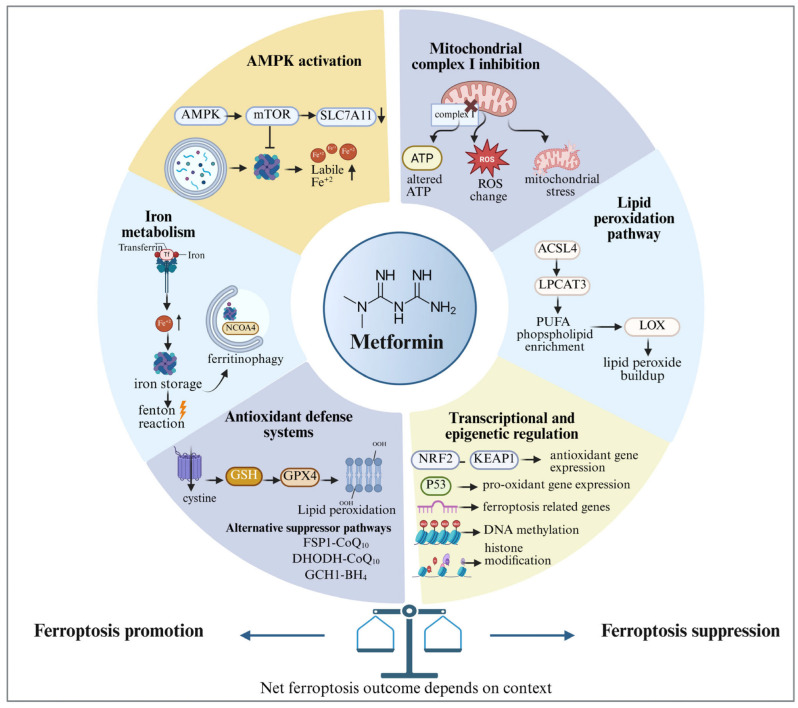
Molecular mechanisms of metformin-mediated ferroptosis modulation. Metformin affects AMPK/mTOR/SLC7A11 signaling, mitochondrial complex I activity, iron metabolism, lipid peroxidation, antioxidant defense systems, and transcriptional or epigenetic regulators. The balance among these modules determines whether ferroptosis is promoted or suppressed. **The colored sectors represent distinct mechanistic pathways involved in metformin-mediated ferroptosis modulation, while the arrows indicate the direction of signaling or regulatory interactions.** Created in BioRender.

The principal molecular mechanisms by which metformin may influence ferroptosis are summarized in Figure 1. Metformin modulates ferroptotic susceptibility through several interconnected regulatory layers, including AMPK-mTOR signaling, inhibition of mitochondrial complex I, iron metabolism, lipid peroxidation pathways, antioxidant defense systems, and transcriptional or epigenetic regulation. These pathways do not operate independently. AMPK activation can alter mTOR activity, system Xc^−^-dependent cystine uptake, autophagy, and ferritinophagy, while mitochondrial complex I inhibition reshapes ATP availability, reactive oxygen species (ROS) generation, and mitochondrial stress responses [16,20]. Simultaneous alterations in iron uptake, ferritin storage, ferroportin-mediated export, and ferritinophagy regulate the pool of redox-active iron available for Fenton chemistry [21]. Lipid peroxidation is further shaped by ACSL4/LPCAT3-dependent PUFA–phospholipid remodeling and by the balance between lipid peroxide generation and detoxification through GPX4, FSP1-CoQ10, DHODH-CoQ10, and GCH1-BH4 systems [22]. Transcriptional regulators such as NRF2, KEAP1, and p53, along with microRNAs and epigenetic modifications, further determine whether metformin exposure promotes or suppresses ferroptosis [23]. Thus, Figure 1 illustrates that the net ferroptotic outcome of metformin treatment is governed by pathway integration rather than by a single dominant mechanism.

The same AMPK-dependent regulatory axis may also confer resistance to ferroptosis by activating NRF2-mediated antioxidant defenses. NRF2 signaling supports cellular energy homeostasis, promotes mitochondrial quality control, and transcriptionally regulates several ferroptosis-relevant protective factors, including GPX4, SLC7A11, ferritin heavy and light chains, heme oxygenase-1 (HO-1), and NAD(P)H quinone dehydrogenase 1 (NQO1) [24]. Upregulation of these targets enhances redox buffering capacity and facilitates iron sequestration, thereby limiting lipid peroxidation and ferroptotic injury [25]. Accordingly, AMPK should not be interpreted as an intrinsically pro- or anti-ferroptotic regulator. Rather, the outcome of AMPK activation depends on the magnitude and duration of signaling, the underlying metabolic state, and the availability of compensatory antioxidant pathways [26,27]. In highly metabolic cancer cells that rely on system Xc^−^-mediated cystine uptake, metformin-induced AMPK activation may suppress mTOR signaling, reduce system Xc^−^ expression, and increase ferroptotic vulnerability [28]. Conversely, in neurons, where oxidative stress tolerance is limited and mitochondrial integrity is critical, AMPK activation may preferentially reinforce NRF2-dependent antioxidant programs and preserve mitochondrial function, thereby protecting against ferroptotic cell death [29]. These divergent outcomes emphasize that the impact of AMPK activation on ferroptosis is highly context-dependent and shaped by cell-specific metabolic and redox backgrounds.

Inhibition of mitochondrial complex I represents another key mechanism by which metformin may modulate ferroptosis. At low or moderate concentrations, often below 1 mM in vitro or within the range achieved by standard clinical dosing in vivo, partial inhibition of complex I may induce a hormetic stress response [30]. Such adaptive mitochondrial stress can activate antioxidant defense programs and promote mitophagy, thereby limiting the accumulation of dysfunctional mitochondria and reducing excessive ROS production [31]. In contrast, higher metformin concentrations, particularly above 2–5 mM in vitro or under supratherapeutic conditions, may overwhelm mitochondrial adaptive capacity, especially during prolonged exposure or in metabolically inflexible cells [32]. Under these conditions, complex I inhibition may contribute to ATP depletion, excessive ROS generation, and disruption of iron–sulfur cluster homeostasis. Impaired iron–sulfur cluster biology may increase the labile iron pool and thereby facilitate ferroptosis-associated lipid peroxidation [33,34]. However, these concentration-dependent thresholds are likely to vary according to cell type, metabolic state, exposure duration, and experimental conditions. Therefore, careful titration of metformin is essential when evaluating its dual pro- and anti-ferroptotic effects.

Iron homeostasis strongly influences this context dependency. Cancer cells differ in their iron metabolism, and their iron dependency increases due to their proliferation and expansion. Transferrin receptor overexpression for iron import, ferritin level dysregulation, and ferroportin downregulation to decrease iron export have been observed in malignant cells [35]. NCOA4 is a cargo receptor that directs ferritin to the lysosome, i.e., ferritinophagy, and therefore increases ferroptosis sensitivity [36]. In cancer cells, metformin-induced autophagy may promote NCOA4-dependent ferritinophagy [37]. This releases stored iron from ferritin and expands the labile iron pool, increasing Fenton chemistry and lipid radical generation. In non-malignant cells with intact NRF2 signaling, metformin may enhance ferritin-mediated iron sequestration and ferroportin-dependent export. This reduces the pool of redox-active iron. The balance between ferritinophagy, ferritin synthesis, iron uptake, and iron export determines the overall effect [38,39].

Lipid peroxidation constitutes the execution phase of ferroptosis. ACSL4 activates arachidonic and adrenic acids, LPCAT3 incorporates the resulting PUFA-CoAs into membrane phospholipids, and subsequent lipoxygenase-dependent or radical-mediated reactions generate lipid hydroperoxides; together, these enzymes shape the PUFA composition of membranes and thereby determine ferroptosis sensitivity [40]. Metformin may influence this process indirectly through AMPK-dependent lipid metabolism, mitochondrial ROS, iron availability, and induction of antioxidant enzymes [38,39,40,41]. Nevertheless, several mechanistic questions remain unresolved. It is still unclear whether metformin directly affects the activity, expression, substrate preference, or post-translational modification of ACSL4, LPCAT3, or lipoxygenases [42]. Advanced lipidomics, activity-based protein profiling, and genetic manipulation (e.g., CRISPR knockout or overexpression models) would help define the contribution of these enzymes to metformin-mediated ferroptosis. A further open question concerns whether the resulting lipid peroxides are detoxified or allowed to accumulate, which depends on GPX4 activity and on parallel ferroptosis-suppressing systems. Because the CoQ oxidoreductase FSP1 acts in parallel with GPX4 to restrain lipid peroxidation, the FSP1-CoQ10 axis may confer ferroptosis resistance when GPX4 function is compromised [43]. Pharmacological inhibition or genetic disruption of FSP1 or GPX4 in the presence of metformin would therefore help clarify how metformin intersects with these control pathways. Transcriptional and epigenetic regulators add an additional layer of complexity: NRF2 promotes antioxidant adaptation, p53 can repress SLC7A11 under specific conditions, and microRNA- or chromatin-level changes may establish more durable ferroptosis phenotypes [44,45]. The relative roles and regulatory hierarchy of these factors during metformin treatment remain to be defined, and integrative transcriptomic and epigenetic profiling following metformin exposure would be informative.

## 3. Context-Dependent Effects Across Cell Types and Disease States

### 3.1. Pro-Ferroptotic Effects in Cancer

The strongest evidence supporting metformin’s pro-ferroptotic potential has emerged from cancer biology. Malignant cells require increased iron availability to sustain DNA synthesis, mitochondrial activity, and rapid proliferation [46]. Owing to oncogenic signaling, mitochondrial remodeling, and heightened biosynthetic demand, cancer cells often operate near an oxidative threshold, accompanied by increased ROS production [47]. This metabolic and redox state creates substantial dependence on system Xc^−^, glutathione (GSH), glutathione peroxidase 4 (GPX4), nuclear factor erythroid 2–related factor 2 (NRF2), and other antioxidant defense systems. Within this vulnerable context, metformin-induced activation of AMP-activated protein kinase (AMPK), suppression of mechanistic target of rapamycin (mTOR), downregulation of SLC7A11, induction of ferritinophagy, and mitochondrial complex I stress may collectively exceed the antioxidant buffering capacity of cancer cells, thereby promoting ferroptotic cell death [15].

In triple-negative breast cancer, metformin has been associated with increased ferroptotic susceptibility through SLC7A11 modulation and enhanced sensitivity to ferroptosis inducers such as erastin and RSL3 [15]. In hepatocellular carcinoma, metformin may facilitate ferroptosis via the AMPK-Mtor-SLC7A11 axis and may act synergistically with sorafenib [48]. Similar vulnerabilities have also been reported in other malignancies. For example, ovarian cancer cells may become particularly sensitive to metformin-induced energy stress, with evidence of both apoptotic and ferroptotic cell death accompanied by glutathione depletion [37]. In acute myeloid leukemia models, metformin-associated ferroptotic responses have been linked to lipidomic remodeling, iron dysregulation, and NRF2-related mechanisms [49]. However, prospective studies correlating metformin exposure with ferroptosis-related biomarkers in clinical cancer cohorts remain necessary [50].

The cancer literature further emphasizes the relevance of ferroptosis resistance mechanisms. NRF2 hyperactivation, KEAP1 loss, FSP1 upregulation, increased GPX4 expression, and metabolic plasticity may all increase the ferroptotic threshold of tumor cells. In tumors characterized by these adaptive features, metformin monotherapy is unlikely to exert sufficient anticancer activity [28]. Nevertheless, metformin may have therapeutic value as a sensitizing agent when combined with system Xc^−^ inhibitors, GPX4 inhibitors, iron-dependent ferroptosis inducers, radiotherapy, or immunotherapy [51,52]. Accordingly, metformin should be considered primarily as a context-dependent ferroptosis sensitizer rather than a universal ferroptosis-inducing anticancer monotherapy.

### 3.2. Anti-Ferroptotic Cytoprotection in Non-Malignant Tissues

In non-malignant tissues, particularly under oxidative or metabolic stress, metformin may exert predominantly cytoprotective and anti-ferroptotic effects. Neurons, cardiomyocytes, hepatocytes, renal tubular epithelial cells, and osteoblasts depend on adaptive stress-response pathways to preserve redox balance and limit oxidative injury. In these cellular contexts, metformin-mediated activation of AMPK–NRF2 signaling may enhance mitochondrial quality control, maintain glutathione-dependent antioxidant capacity, stabilize iron homeostasis, and reduce lipid peroxide accumulation [11].

Neurodegenerative and ischemic brain injury models provide important examples of this protective paradigm. Iron accumulation, mitochondrial dysfunction, and lipid peroxidation contribute to the pathogenesis of Parkinson’s disease, Alzheimer’s disease, stroke, and traumatic brain injury [53,54]. In preclinical models of cerebral ischemia–reperfusion injury, metformin has been associated with AMPK–NRF2 activation, enhanced mitophagy, and attenuation of neuronal ferroptosis [17]. Together with preclinical studies, several clinical studies investigating the effects of metformin in patients with Alzheimer’s disease (AD), Parkinson’s disease (PD), and multiple sclerosis are currently ongoing [55,56,57]. Several retrospective cohort studies and meta-analyses in patients with type 2 diabetes have reported that metformin exposure is associated with a lower risk of dementia or cognitive decline compared with non-use or alternative antidiabetic therapies [58,59,60,61]. These population-level findings are biologically compatible with the ability of metformin to improve insulin sensitivity, reduce systemic inflammation, regulate mitochondrial metabolism, and activate AMPK-related adaptive stress responses. However, these studies do not directly demonstrate suppression of neuronal ferroptosis because most real-world cohorts lack ferroptosis-specific biomarkers, such as brain lipid peroxidation products, labile iron pool measurements, GPX4 activity, ACSL4/LPCAT3 expression, or ferroptosis-sensitive neuroimaging markers. In addition, confounding by diabetes severity, renal function, vitamin B12 deficiency, vascular comorbidities, treatment duration, and healthy-user bias may influence the observed association between metformin and dementia outcomes [60,61,62]. Therefore, current clinical data should be viewed as supportive but not definitive evidence for metformin-mediated ferroptosis inhibition. Future studies should integrate longitudinal cognitive outcomes with pharmacodynamic markers of oxidative lipid damage, iron metabolism, mitochondrial dysfunction, and neuroinflammation to determine whether metformin exposure is truly associated with reduced neuroferroptotic injury in humans.

Cardiovascular models further support metformin’s anti-ferroptotic potential in non-malignant tissues. Doxorubicin-induced cardiotoxicity is characterized by mitochondrial ROS generation, iron dysregulation, lipid peroxidation, and ferroptosis-mediated cardiomyocyte injury [63]. In preclinical studies, metformin has been shown to preserve mitochondrial function, activate AMPK-dependent protective signaling, and reduce lipid peroxide accumulation. Similarly, myocardial ischemia–reperfusion injury involves ferroptosis-associated mitochondrial damage and iron-mediated oxidative stress, both of which may be mitigated by metformin through AMPK–NRF2 activation and improved mitochondrial homeostasis [64].

Metabolic and tissue-injury models further extend this cytoprotective framework. In non-alcoholic fatty liver disease (NAFLD) and non-alcoholic steatohepatitis (NASH), hepatocyte ferroptosis contributes to inflammation, lipotoxic injury, and disease progression, whereas metformin-mediated improvements in redox balance, lipid metabolism, and mitochondrial function may reduce ferroptosis-associated hepatic damage [65,66]. In models of intestinal ischemia–reperfusion injury and diabetic osteoporosis, metformin has also been proposed to suppress ferroptosis through AMPK activation, reinforcement of antioxidant defenses, and maintenance of iron homeostasis. However, these findings remain largely preclinical and require further validation in disease-relevant clinical cohorts [16,66].

### 3.3. Dose, Exposure Duration, and Tissue Distribution

Dose, exposure duration, and tissue distribution are critical determinants of the biological effects of metformin on ferroptosis. Therapeutic plasma concentrations achieved during standard clinical use typically remain in the micromolar range, approximately 10–40 μM, whereas many in vitro studies employ millimolar concentrations [12,67]. This pharmacological discrepancy may substantially influence both the direction and magnitude of ferroptotic or cytoprotective outcomes. Therefore, findings obtained under high-dose experimental conditions should be interpreted cautiously when extrapolated to clinical settings.

Tissue-specific accumulation may partly explain why systemic plasma concentrations do not fully reflect local biological activity. Higher intracellular or regional metformin exposure may occur in tissues with substantial expression of organic cation transporters, such as the liver, intestine, or tumors expressing organic cation transporter 1 (OCT1) [68]. Beyond transporter expression, germline pharmacogenetic variation may further contribute to interindividual differences in metformin tissue exposure and therapeutic response. SLC22A1, which encodes OCT1, is highly polymorphic, and reduced-function variants such as R61C, G401S, M420del/420del, and G465R have been associated with altered metformin uptake or pharmacodynamic response in experimental and clinical studies [69]. Because OCT1 is a major determinant of hepatic metformin entry, individuals carrying reduced-function SLC22A1 alleles may exhibit lower intrahepatic drug accumulation and weaker pathway engagement despite similar systemic dosing [70]. Conversely, preserved or high OCT1 function may favor greater hepatic exposure and stronger AMPK-related pharmacodynamic effects [71]. This issue is particularly relevant to liver disease and hepatocellular carcinoma, where hepatic OCT1 activity may influence both metabolic and ferroptosis-related outcomes [72].

Transporter heterogeneity may also affect oncology applications. Tumors with higher expression or functional activity of organic cation transporters may accumulate more metformin and may therefore be more likely to show AMPK activation, mitochondrial complex I stress, SLC7A11 modulation, and ferroptosis sensitization [73]. In contrast, tumors with low transporter expression or reduced-function transporter genotypes may fail to achieve sufficient intracellular metformin concentrations for ferroptosis-related effects, even when circulating drug levels are clinically adequate [74]. Population differences in SLC22A1 variant frequencies further suggest that transporter genotype could contribute to interethnic variability in metformin efficacy and tolerability [75]. In addition, efflux transporters such as MATE1/SLC47A1 and MATE2/SLC47A2 may modify the intracellular retention and systemic disposition of metformin [76]. Therefore, future clinical studies should consider OCT/MATE transporter expression, SLC22A1 genotype, and, where feasible, SLC47A1/SLC47A2 variation as part of biomarker-guided stratification. Such profiling may help distinguish patients with likely adequate tumor or hepatic metformin exposure from those in whom limited intracellular accumulation may reduce the likelihood of modulating ferroptosis. In these settings, local metformin accumulation may impose greater metabolic stress than would be predicted from circulating plasma levels alone. Exposure duration represents another key variable. Acute metformin treatment may predominantly activate adaptive stress-response pathways, whereas prolonged exposure may progressively reshape cellular metabolism, iron handling, mitochondrial function, and antioxidant capacity.

**Figure 2:**
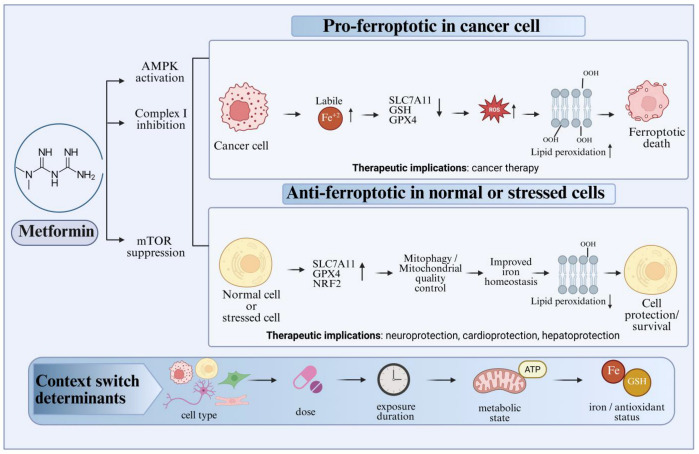
Context-dependent effects of metformin on ferroptosis. Cancer cells with high basal ROS, iron demand, and metabolic stress may undergo pro-ferroptotic responses, whereas normal or stressed non-malignant cells may activate NRF2, GPX4/SLC7A11, mitophagy, and iron homeostasis to reduce lipid peroxidation and support survival. Created in BioRender.

As illustrated in Figure 2, the divergent effects of metformin on ferroptosis can be conceptualized as a context-dependent biological switch. In cancer cells, high basal ROS production, increased iron demand, metabolic stress, and dependence on system Xc^−^/GPX4-mediated antioxidant defenses may favor a pro-ferroptotic response. By contrast, in normal or stressed non-malignant cells, intact adaptive stress responses, lower baseline oxidative burden, and greater capacity for AMPK–NRF2-mediated cytoprotection may shift the outcome toward ferroptosis suppression and cell survival. This switch is shaped by several interacting determinants, including cell type, metformin dose, exposure duration, metabolic state, iron availability, and antioxidant capacity.

Consequently, dose, timing, transporter expression, and tissue context should be explicitly considered when evaluating the dual pro- and anti-ferroptotic effects of metformin. Building on the conceptual framework presented in Figure 2, key methodological and translational factors that should be considered when designing or interpreting metformin–ferroptosis studies are summarized in Table 1.

### 3.4. Quantitative Pharmacokinetic Gap Between Preclinical and Clinical Metformin Exposure

A major translational limitation in metformin–ferroptosis research is the quantitative mismatch between concentrations commonly used in vitro and those achieved during clinical treatment. Standard oral metformin dosing in humans, usually 1–2 g/day, produces systemic plasma concentrations in the micromolar range, most commonly approximately 10–40 μM [12,67,68]. After oral administration, portal venous and hepatic exposure may be higher because metformin is absorbed from the intestine and undergoes first-pass hepatic uptake. Portal concentrations have been estimated at approximately 50–60 μM, and hepatic exposure may reach approximately 60–90 μM or, in experimental estimates, around 50–100 μM [77,78]. Nevertheless, these values remain far below the millimolar concentrations used in many cell-culture studies.

This concentration gap is substantial. A dose of 1 mM corresponds to 1000 μM, which is approximately 25–100 times higher than typical systemic clinical plasma concentrations of 10–40 μM and approximately 10–20 times higher than estimated hepatic exposure of 50–100 μM. In vitro concentrations of 5–10 mM therefore represent approximately 125–1000-fold higher exposure than clinical plasma levels and approximately 50–200-fold higher exposure than estimated hepatic levels [67,79,80]. This distinction is biologically important because several mechanisms frequently attributed to metformin, particularly robust mitochondrial complex I inhibition, ATP depletion, altered adenine nucleotide ratios, and strong energetic stress, are most consistently observed at suprapharmacological millimolar concentrations [11]. By contrast, clinically relevant micromolar concentrations may preferentially produce subtler effects on hepatic redox balance, substrate-selective gluconeogenesis, transporter-dependent tissue accumulation, and adaptive stress responses [12,77,81].

Therefore, the interpretation of metformin-induced ferroptosis requires explicit concentration stratification. Ferroptosis-promoting effects observed at 1–10 mM in vitro should not be directly extrapolated to patients unless intracellular or tissue-level exposure is measured and shown to be pharmacologically plausible. Future studies should include clinically relevant concentration ranges, such as 10–100 μM for systemic or hepatic exposure modeling, alongside higher concentrations only when justified as supratherapeutic stress models [12,67,79]. In cancer models, additional caution is required because tumor metformin accumulation may depend on OCT/MATE transporter expression, tumor perfusion, metabolic state, and exposure duration. Accordingly, metformin–ferroptosis studies should report extracellular dose, exposure duration, estimated intracellular or tissue concentration where possible, transporter status, and whether the observed ferroptotic phenotype occurs within a clinically plausible exposure window.

In addition to these methodological considerations, biomarker selection is essential for distinguishing ferroptosis induction, ferroptosis resistance, and cytoprotective adaptation. Candidate biomarkers that may help evaluate metformin-mediated ferroptosis modulation are listed in Table 2.

## 4. Therapeutic Implications and Clinical Translation

The therapeutic relevance of metformin in ferroptosis biology arises from its capacity to modulate ferroptotic susceptibility in opposite directions depending on disease context. In oncology, metformin may intensify pro-ferroptotic pressure in metabolically vulnerable tumor cells. In degenerative, cardiovascular, and metabolic disorders, however, metformin may preferentially promote adaptive anti-ferroptotic responses that protect non-malignant tissues. A central translational challenge is therefore to determine which biological mode predominates in a given patient, tissue, metabolic state, and therapeutic combination.

**Figure 3:**
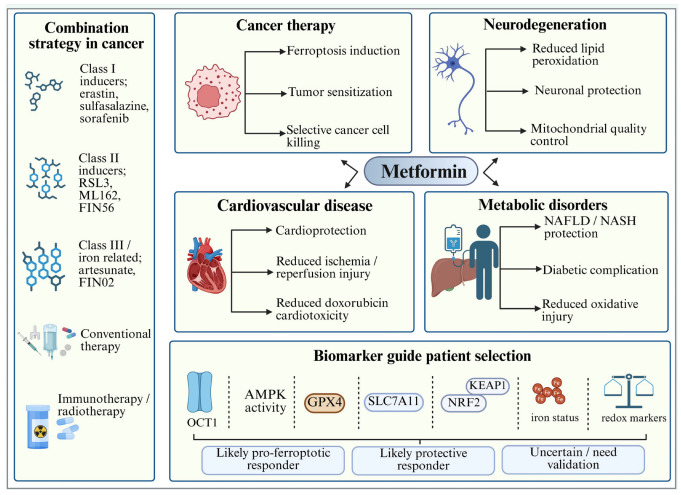
Therapeutic implications of metformin-mediated ferroptosis modulation. In oncology, metformin may be combined with ferroptosis inducers, conventional therapy, radiotherapy, or immunotherapy. In neurodegeneration, cardiovascular disease, and metabolic disorders, the same drug may contribute to anti-ferroptotic tissue protection. Biomarker-guided patient selection is central to translation. Created in BioRender.

This translational framework is summarized in Figure 3. In oncology, metformin may be positioned as a ferroptosis-sensitizing agent within rational combination strategies, including system Xc^−^-targeting inducers, GPX4-directed ferroptosis inducers, iron-dependent agents, conventional chemotherapy, radiotherapy, and immunotherapy. In contrast, in neurodegenerative, cardiovascular, and metabolic disorders, metformin may preferentially support anti-ferroptotic cytoprotection by reducing lipid peroxidation, preserving mitochondrial quality control, limiting ischemia–reperfusion injury, and improving oxidative stress-related tissue damage. Figure 3 also emphasizes that clinical translation should not rely on disease category alone but should incorporate biomarker-guided patient selection based on transporter status, AMPK pathway engagement, GPX4 and SLC7A11 expression, NRF2/KEAP1 status, iron parameters, and redox markers. This approach may help distinguish likely pro-ferroptotic responders, likely protective responders, and patients in whom the ferroptosis-related effect of metformin remains uncertain and requires further validation.

In cancer therapy, metformin may be most appropriately positioned within biomarker-guided combination strategies rather than as a standalone ferroptosis-inducing agent. When combined with system Xc^−^-targeting ferroptosis inducers such as erastin, sulfasalazine, or sorafenib, metformin may reinforce cystine deprivation by suppressing SLC7A11 expression and reducing glutathione (GSH) availability [15]. In combination with GPX4-directed agents such as RSL3, ML162, or FIN56, metformin may increase lipid hydroperoxide accumulation and weaken the antioxidant reserve required for GPX4-dependent detoxification [82]. Similarly, when paired with iron-dependent ferroptosis inducers such as artesunate or FINO2, metformin-associated ferritinophagy and mitochondrial stress may increase the labile iron pool and amplify lipid peroxidation driven by iron-dependent redox cycling [83].

Importantly, ferroptosis inducers should not be considered a uniform therapeutic class when combined with metformin because their toxicity profiles, selectivity, and translational maturity differ substantially [15]. System Xc^−^-targeting agents such as erastin and sulfasalazine may enhance cystine deprivation and glutathione depletion; however, erastin remains mainly a preclinical tool compound with limited in vivo drug-like performance, whereas sulfasalazine is clinically available but may be associated with gastrointestinal intolerance, hypersensitivity reactions, hepatic effects, and hematologic toxicity [84,85]. Sorafenib, although clinically approved and mechanistically linked to ferroptosis in hepatocellular carcinoma, is a multikinase inhibitor rather than a selective ferroptosis inducer, and its combination with metformin should therefore take into account dermatologic toxicity, diarrhea, hypertension, fatigue, and hepatic reserve [86]. In contrast, direct GPX4- or lipid peroxide detoxification-targeting compounds such as RSL3, ML162, FIN56, and FINO2 may impose stronger ferroptotic pressure, but most remain preclinical and may theoretically increase the risk of normal-tissue lipid peroxidation, particularly in metabolically active organs such as the heart, kidney, liver, and nervous system [87,88]. Therefore, metformin-based ferroptosis-sensitizing strategies should be evaluated by inducer class, tumor context, expected tissue exposure, renal and hepatic function, and baseline redox vulnerability, rather than assuming a shared safety profile across all ferroptosis-inducing agents [89].

The rationale for combining metformin with conventional anticancer therapies is also mechanistically plausible. Platinum agents can deplete GSH and induce oxidative damage; radiotherapy generates ROS and may suppress SLC7A11-dependent antioxidant defense; and immunotherapy may promote tumor ferroptosis through CD8+ T cell-mediated inflammatory signals and lipid oxidation [90]. In these settings, metformin could potentially amplify tumor-specific metabolic and redox stress while also modulating the tumor microenvironment and T cell metabolism. However, such combinations require careful contextual evaluation. In particular, regimens involving doxorubicin warrant caution because anthracycline-induced cardiotoxicity itself involves ferroptosis-associated mitochondrial injury, iron dysregulation, and lipid peroxidation [91]. Tissue-specific scheduling, dose optimization, and safety monitoring are therefore essential when attempting to exploit ferroptosis therapeutically.

Beyond oncology, the anti-ferroptotic and cytoprotective properties of metformin may be therapeutically relevant in ferroptosis-associated tissue injury. In neurodegenerative diseases, metformin may reduce lipid peroxidation, enhance mitochondrial quality control, and strengthen neuronal antioxidant defenses [92]. In cardiovascular disease, metformin may attenuate ischemia–reperfusion injury and potentially mitigate anthracycline-associated cardiotoxicity [64,93]. In metabolic liver disease, including NAFLD and NASH, improvements in redox homeostasis, lipid metabolism, and mitochondrial function may reduce hepatocyte ferroptosis and associated inflammatory injury.

A biomarker-guided translational framework is therefore essential. Candidate biomarkers include OCT1 and related transporter profiles, phospho-AMPK, GPX4, SLC7A11, NRF2/KEAP1 status, iron-related parameters, lipid peroxidation markers, and GSH/GSSG ratios (Table 2). Future clinical studies should move beyond simple metformin add-on designs and incorporate baseline ferroptosis phenotyping, pharmacodynamic sampling, transporter profiling, tissue-specific safety monitoring, and rational drug combinations matched to tumor or tissue context. Without such stratification, heterogeneous patient populations may include individuals in whom metformin drives divergent ferroptosis-related outcomes, thereby diluting efficacy signals and obscuring clinically meaningful therapeutic effects.

## 5. Open Questions and Future Directions

**Figure 4:**
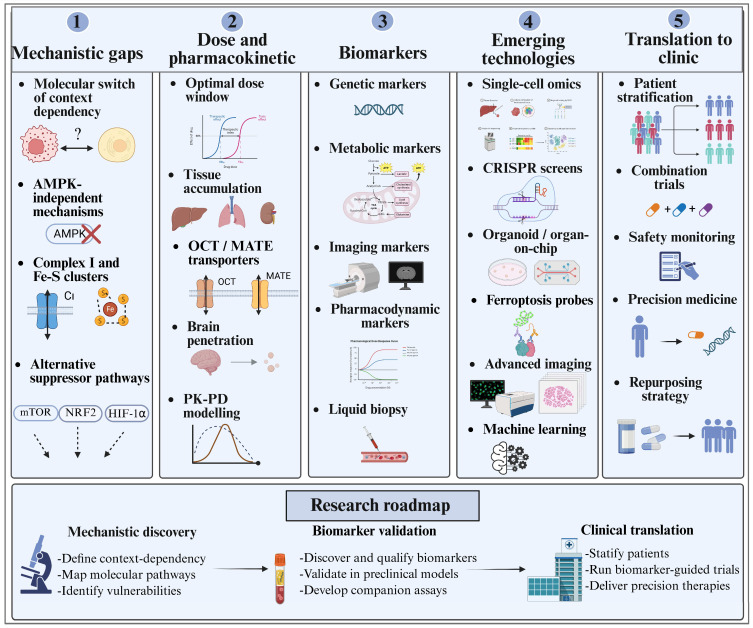
Open questions and future directions. The figure summarizes key priorities for advancing metformin-ferroptosis research, including identification of context-dependent molecular switches, clarification of dose and pharmacokinetic parameters, biomarker validation, application of emerging technologies, and translation into biomarker-guided clinical studies. A staged roadmap from mechanistic discovery to biomarker validation and clinical translation may help prioritize future research. Created in BioRender.

The major unresolved questions and future research priorities in metformin-mediated modulation of ferroptosis are summarized in Figure 4. Current evidence indicates that the biological outcome of metformin treatment is governed by several interacting layers, including mechanistic context dependency, dose and pharmacokinetic variability, biomarker availability, and disease-specific translational requirements. Key mechanistic gaps include identifying molecular switches that determine pro- versus anti-ferroptotic responses, assessing the contribution of AMPK-independent pathways, elucidating the role of mitochondrial complex I and iron–sulfur cluster biology, and investigating the involvement of alternative ferroptosis-suppressing systems. From a translational perspective, clinically relevant dose windows, tissue accumulation, OCT/MATE transporter activity, blood–brain barrier penetration, and pharmacokinetic–pharmacodynamic relationships require further clarification. Figure 4 also highlights the need for biomarker discovery and validation using genetic, metabolic, imaging, pharmacodynamic, and liquid biopsy-based approaches. Emerging technologies, including single-cell omics, CRISPR screening, patient-derived organoids, organ-on-chip platforms, ferroptosis probes, advanced imaging, and machine learning, may help connect mechanistic discovery with biomarker-guided clinical translation. Overall, this roadmap supports a staged strategy that progresses from mechanistic discovery to biomarker validation and, ultimately, to precision clinical application.

A central unresolved issue concerns the identity of the molecular switch that determines whether metformin promotes or inhibits ferroptosis in cells. This regulatory mechanism is unlikely to be controlled by a single pathway; instead, it likely results from the quantitative interplay among iron availability, lipid peroxide generation, GPX4 activity, system Xc^−^ function, NRF2 responsiveness, mitochondrial performance, and metabolic adaptability. Systems biology approaches that integrate transcriptomics, proteomics, lipidomics, and metabolomics may be necessary to delineate these thresholds. To directly investigate candidate molecular switches, future work could employ CRISPR-Cas9 screening in isogenic cell lines exposed to metformin and ferroptosis inducers under varying iron and antioxidant conditions. Comparative studies using organoid models or co-culture systems that represent relevant tissue and disease contexts may help clarify how cellular heterogeneity shapes metformin’s effects. Time-resolved multi-omics analysis before and after metformin exposure, combined with functional rescue experiments targeting GPX4, system Xc^−^, or NRF2, would further define the regulatory circuitry involved.

Mechanisms independent of AMPK warrant further investigation. While AMPK is a key mediator of metformin pharmacology, metformin can modulate complex I, lysosomal signaling, redox balance, and cellular metabolism outside canonical AMPK pathways. Distinguishing AMPK-dependent from AMPK-independent effects on ferroptosis will require AMPK-null models, rescue experiments, pharmacological controls, and comparisons with structurally unrelated inhibitors of complex I. Clarifying these mechanisms has important clinical relevance, as it could inform patient stratification and optimize therapeutic targeting. For example, if AMPK-independent pathways predominate in certain contexts or patient subsets, treatment strategies could be tailored accordingly, potentially enhancing efficacy and minimizing off-target effects.

Pharmacokinetic properties and tissue distribution represent significant translational challenges. In vitro concentrations often exceed clinically achievable plasma levels, and local concentrations in the gut, liver, and tumors may differ markedly from systemic exposure. Organic cation transporters (OCTs) and multidrug and toxin extrusion (MATE) transporters regulate metformin uptake and efflux, and genetic or disease-related variations may contribute to heterogeneous responses [94]. For neurodegenerative indications, restricted blood–brain barrier penetration constitutes an additional limitation [95]. To address these challenges, strategies such as dose optimization based on pharmacokinetic modeling, modulation of transporter activity to enhance tissue-specific drug delivery, and the development of alternative routes of administration, including nanoparticles, prodrugs, and targeted delivery systems, should be explored. These approaches may help to better align preclinical findings with clinically relevant exposures and support the translation of mechanistic insights into therapeutic applications.

Comprehensive biomarker validation should follow a staged approach. Initially, candidate biomarkers must be assessed in preclinical models to evaluate reproducibility and their correlation with treatment response. Subsequently, promising markers should be evaluated in retrospective and prospective clinical cohorts to determine sensitivity, specificity, and predictive value. The application of single-cell omics, CRISPR screening, patient-derived organoids, organ-on-chip platforms, ferroptosis probes, and advanced imaging technologies may expedite this process by connecting mechanistic insights to clinically actionable signatures. Ultimately, validated biomarkers could be integrated into patient selection criteria or used as pharmacodynamic and clinical endpoints in future trials, thereby enhancing stratification, monitoring treatment responses, and improving the overall translational impact of metformin-based interventions.

## 6. Conclusions

Metformin should be regarded as a context-dependent modulator of ferroptosis rather than as a uniform inducer or suppressor of this cell death pathway. Through AMPK activation, mitochondrial complex I inhibition, iron remodeling, lipid metabolic regulation, and modulation of antioxidant defense systems, metformin may either increase ferroptotic susceptibility or promote cytoprotection. The final biological outcome is shaped by cellular background, disease state, metabolic flexibility, iron availability, and compensatory antioxidant capacity.

In cancer cells, elevated iron demand, increased ROS production, antioxidant dependence, and metabolic stress may create a vulnerable state in which metformin enhances pro-ferroptotic pressure, particularly when combined with ferroptosis inducers, chemotherapy, radiotherapy, or immunotherapy. Conversely, in normal or stressed non-malignant tissues, intact NRF2 signaling, mitophagy, iron sequestration, and GPX4-dependent lipid peroxide detoxification may shift the response toward anti-ferroptotic cytoprotection.

In the short term, research with clinical applicability should prioritize enhancing the translational reliability of metformin–ferroptosis investigations. Key objectives include evaluating both clinically relevant micromolar and mechanistic millimolar metformin concentrations, standardizing ferroptosis endpoints, systematically reporting exposure duration and culture conditions, quantifying OCT/MATE transporter expression, and incorporating pharmacodynamic markers such as phospho-AMPK, GPX4, SLC7A11, GSH/GSSG ratio, lipid peroxidation products, and iron-related parameters. Analyses of retrospective clinical cohorts and real-world datasets can further elucidate associations between metformin exposure and disease outcomes in oncology, dementia, cardiovascular injury, and metabolic liver disease. However, it is important to acknowledge that such studies cannot establish causality specific to ferroptosis in the absence of mechanistic biomarkers.

In the long term, research should elucidate the molecular determinants governing whether metformin promotes or suppresses ferroptosis in specific biological contexts. Achieving this objective will require integrating time-resolved multi-omics, lipidomics, CRISPR-based genetic screens, patient-derived organoids, organ-on-chip systems, advanced ferroptosis imaging, and prospective biomarker-guided clinical trials. Special emphasis should be placed on AMPK-independent mechanisms, mitochondrial complex I function, NCOA4-dependent ferritinophagy, ACSL4/LPCAT3-mediated lipid remodeling, NRF2/KEAP1 status, and transporter-mediated tissue exposure. These strategies may ultimately enable the precision application of metformin to enhance ferroptosis in selected tumors or to mitigate ferroptotic injury in degenerative, cardiovascular, and metabolic diseases.

Despite these opportunities, several limitations currently hinder the direct clinical implementation of metformin as a ferroptosis-targeted therapeutic agent. First, most available evidence is preclinical and derived from cell culture or animal models, while prospective human studies directly linking metformin exposure to ferroptosis-specific outcomes are lacking. Second, many in vitro studies employ millimolar metformin concentrations that exceed typical clinical plasma levels by orders of magnitude, complicating pharmacological extrapolation unless intracellular accumulation, tissue exposure, and transporter expression are quantified [96]. Third, no validated clinical biomarker distinguishes ferroptosis from ROS, inflammatory markers, or mitochondrial dysfunction [97]. Commonly used markers such as malondialdehyde, 4-hydroxynonenal, GSH/GSSG ratio, ferritin, GPX4, SLC7A11, and ACSL4 are informative but not individually specific to ferroptosis [98]. Fourth, patient heterogeneity in renal function, diabetes severity, iron status, tumor genotype, NRF2/KEAP1 activity, OCT/MATE transporter expression, and concomitant therapies may significantly influence metformin exposure and ferroptosis sensitivity [99]. Finally, combination strategies involving metformin with ferroptosis inducers, chemotherapy, radiotherapy, or immunotherapy may result in tissue-specific toxicities, particularly in organs susceptible to lipid peroxidation, such as the heart, liver, kidney, and nervous system. Consequently, metformin-based ferroptosis modulation should not yet be considered suitable for broad clinical application; rather, it should be advanced through biomarker-guided, dose-justified, tissue-specific, and safety-monitored translational studies.

## Figures and Tables

**Table 1:** Translational considerations for metformin-ferroptosis studies [12,67,68].

Parameter	Preclinical Issue	Clinical Relevance	Recommended Reporting
Metformin concentration	Often 1–10 mM in vitro	Clinical plasma: 10–40 μM; hepatic exposure may be higher	Report concentration and justify clinical relevance
Exposure duration	Acute exposure may differ from chronic adaptation	Patients usually receive chronic daily dosing	Specify duration and consider adaptive responses
OCT/MATE transporter status	Variable expression across cell lines	Genetic polymorphisms and tissue expression affect uptake	Measure transporter expression or genotype when possible
Cell type/tissue context	Immortalized cell lines may not recapitulate tissue complexity	Tumor heterogeneity and stromal interactions influence response	Use patient-derived models, organoids or co-cultures
Ferroptosis endpoints	Single assays are insufficient	No universally validated clinical ferroptosis biomarker	Report multiple endpoints: lipid peroxidation, iron and GSH/GSSG
Combination partner	Synergy may be tested at high doses	Clinical dosing and toxicity profiles may differ	Test clinically relevant dose combinations
Safety monitoring	Off-target toxicity may be missed preclinically	Cardiac, hepatic and metabolic toxicity are clinically relevant	Monitor tissue-specific toxicity and redox-active co-treatments

**Table 2:** Candidate biomarkers for metformin-mediated ferroptosis modulation. Candidate biomarkers were selected based on their established relevance to metformin uptake and pharmacodynamic signaling, system Xc^−^/GSH/GPX4-dependent antioxidant defense, NRF2/KEAP1-mediated stress adaptation, p53-associated ferroptosis regulation, iron handling, ferritin biology, lipid peroxidation, and ferroptosis sensitivity or resistance [1,3,4,5,11,12,15,18,24,27,28,39,67].

Biomarker	Biological Meaning	Sample Type	Current Validation Level	Use in Cancer/Cytoprotection
OCT1 (SLC22A1)	Metformin uptake	Tumor tissue, IHC/mRNA; blood genotype	Investigational for ferroptosis; established relevance to metformin PK	May indicate tumor or tissue exposure
Phospho-AMPK (Thr172)	Pathway engagement	Tissue IHC/western; blood limited	Pharmacodynamic marker	Confirms AMPK activation
GPX4	Ferroptosis resistance	Tissue IHC/western	Preclinical; not clinically validated for this use	High expression may indicate resistance; low expression may indicate vulnerability
SLC7A11 (xCT)	Cystine uptake and GSH synthesis	Tissue IHC/mRNA	Preclinical; cancer-relevant	High expression may indicate system Xc^−^ dependence/resistance
NRF2/KEAP1	Antioxidant response	Sequencing/IHC	Cancer-relevant; not validated for metformin-ferroptosis selection	Mutations may indicate resistance; activation may indicate protection
p53 status	Ferroptosis sensitivity	Sequencing/IHC	Clinically used in oncology but context-dependent here	Wild-type p53 may enhance ferroptosis in selected contexts
Labile iron	Redox-active iron	Tissue probes; blood limited	Investigational and technically challenging	High LIP may indicate ferroptosis vulnerability
4-HNE, MDA	Lipid peroxidation products	Tissue or plasma	Non-specific supportive markers	Elevated levels suggest oxidative lipid damage but are not ferroptosis-specific
GSH/GSSG ratio	Redox state	Blood or tissue	Measurable but not ferroptosis-specific	Low GSH/GSSG may indicate vulnerability
Ferritin H/L chains	Iron storage	Blood; tissue IHC	Clinically useful for iron status but context-dependent	May reflect iron sequestration or inflammation

Abbreviations: IHC, immunohistochemistry; PK, pharmacokinetics; GSH, glutathione; GSSG, oxidized glutathione; 4-HNE, 4-hydroxynonenal; MDA, malondialdehyde.

## Data Availability

No new datasets were generated or analyzed for this review.

## References

[1] Stockwell B.R. (2022). Ferroptosis Turns 10: Emerging Mechanisms, Physiological Functions, and Therapeutic Applications. Cell.

[2] Chen X., Kang R., Kroemer G., Tang D. (2024). Broadening Horizons: The Role of Ferroptosis in Cancer. Nat. Rev. Clin. Oncol..

[3] Dai E., Chen X., Linkermann A., Jiang X., Kang R., Kagan V.E., Bayir H., Yang W.S., Garcia-Saez A.J., Ioannou M.S. (2024). A Guideline on the Molecular Ecosystem Regulating Ferroptosis. Nat. Cell Biol..

[4] Li F., Long H., Zhou Z., Luo H., Xu S., Gao L. (2022). System Xc−/GSH/GPX4 Axis: An Important Antioxidant System for the Ferroptosis in Drug-Resistant Solid Tumor Therapy. Front. Pharmacol..

[5] Galy B., Conrad M., Muckenthaler M.U. (2024). Mechanisms Controlling Cellular and Systemic Iron Homeostasis. Nat. Rev. Mol. Cell Biol..

[6] Cui J., Wang Y., Tian X., Miao Y., Ma L., Zhang C., Xu X., Wang J., Fang W., Zhang X. (2023). LPCAT3 Is Transcriptionally Regulated by YAP/ZEB/EP300 and Collaborates with ACSL4 and YAP to Determine Ferroptosis Sensitivity. Antioxid. Redox Signal..

[7] Ma T., Du J., Zhang Y., Wang Y., Wang B., Zhang T. (2022). GPX4-Independent Ferroptosis—A New Strategy in Disease’s Therapy. Cell Death Discov..

[8] Mao C., Liu X., Zhang Y., Lei G., Yan Y., Lee H., Koppula P., Wu S., Zhuang L., Fang B. (2021). DHODH-Mediated Ferroptosis Defence Is a Targetable Vulnerability in Cancer. Nature.

[9] Mishima E., Ito J., Wu Z., Nakamura T., Wahida A., Doll S., Tonnus W., Nepachalovich P., Eggenhofer E., Aldrovandi M. (2022). A Non-Canonical Vitamin K Cycle Is a Potent Ferroptosis Suppressor. Nature.

[10] Freitas F.P., Alborzinia H., Dos Santos A.F., Nepachalovich P., Pedrera L., Zilka O., Inague A., Klein C., Aroua N., Kaushal K. (2024). 7-Dehydrocholesterol Is an Endogenous Suppressor of Ferroptosis. Nature.

[11] Foretz M., Guigas B., Viollet B. (2023). Metformin: Update on Mechanisms of Action and Repurposing Potential. Nat. Rev. Endocrinol..

[12] LaMoia T.E., Shulman G.I. (2021). Cellular and Molecular Mechanisms of Metformin Action. Endocr. Rev..

[13] Zhu H., Jia Z., Li Y.R., Danelisen I. (2023). Molecular Mechanisms of Action of Metformin: Latest Advances and Therapeutic Implications. Clin. Exp. Med..

[14] Li Z., Cui C., Xu L., Ding M., Wang Y. (2024). Metformin Suppresses Metabolic Dysfunction-Associated Fatty Liver Disease by Ferroptosis and Apoptosis via Activation of Oxidative Stress. Free Radic. Res..

[15] Yang J., Zhou Y., Xie S., Wang J., Li Z., Chen L., Mao M., Chen C., Huang A., Chen Y. (2021). Metformin Induces Ferroptosis by Inhibiting UFMylation of SLC7A11 in Breast Cancer. J. Exp. Clin. Cancer Res..

[16] Liu Y., Fu Z., Wang X., Yang Q., Liu S., Zhu D. (2025). Metformin Attenuates Diabetic Osteoporosis by Suppressing Ferroptosis via the AMPK/Nrf2 Pathway. Front. Pharmacol..

[17] Wang Z., Wu Z., Xie Z., Zhou W., Li M. (2022). Metformin Attenuates Ferroptosis and Promotes Functional Recovery of Spinal Cord Injury. World Neurosurg..

[18] Song X., Zhu S., Chen P., Hou W., Wen Q., Liu J., Xie Y., Liu J., Klionsky D.J., Kroemer G. (2018). AMPK-Mediated BECN1 Phosphorylation Promotes Ferroptosis by Directly Blocking System Xc-Activity. Curr. Biol..

[19] Tang D., Chen X., Kang R., Kroemer G. (2021). Ferroptosis: Molecular Mechanisms and Health Implications. Cell Res..

[20] Liao H., Wang Y., Zou L., Fan Y., Wang X., Tu X., Zhu Q., Wang J., Liu X., Dong C. (2024). Relationship of MTORC1 and Ferroptosis in Tumors. Discov. Oncol..

[21] Zhang X., Xiang Y., Wang Q., Bai X., Meng D., Wu J., Sun K., Zhang L., Qiang R., Liu W. (2025). Regulation of Iron Metabolism in Ferroptosis: From Mechanism Research to Clinical Translation. J. Pharm. Anal..

[22] Jiang Y., Zhang M., Sun M. (2025). ACSL4 at the Helm of the Lipid Peroxidation Ship: A Deep-Sea Exploration towards Ferroptosis. Front. Pharmacol..

[23] Wang Y., Hu J., Wu S., Fleishman J.S., Li Y., Xu Y., Zou W., Wang J., Feng Y., Chen J. (2023). Targeting Epigenetic and Posttranslational Modifications Regulating Ferroptosis for the Treatment of Diseases. Signal Transduct. Target. Ther..

[24] Xiang Y., Song X., Long D. (2024). Ferroptosis Regulation through Nrf2 and Implications for Neurodegenerative Diseases. Arch. Toxicol..

[25] Ercin N., Beker M., Besli N., Celik U. (2026). Proteostasis Driven Redox Adaptation in Ferroptosis: The P62-Keap1-Nrf2 Axis. Prog. Biophys. Mol. Biol..

[26] Lee H., Zandkarimi F., Zhang Y., Meena J.K., Kim J., Zhuang L., Tyagi S., Ma L., Westbrook T.F., Steinberg G.R. (2020). Energy-Stress-Mediated AMPK Activation Inhibits Ferroptosis. Nat. Cell Biol..

[27] Anandhan A., Dodson M., Schmidlin C.J., Liu P., Zhang D.D. (2020). Breakdown of an Ironclad Defense System: The Critical Role of NRF2 in Mediating Ferroptosis. Cell Chem. Biol..

[28] Deng C., Xiong L., Chen Y., Wu K., Wu J. (2023). Metformin Induces Ferroptosis through the Nrf2/HO-1 Signaling in Lung Cancer. BMC Pulm. Med..

[29] Brandes M.S., Gray N.E. (2020). NRF2 as a Therapeutic Target in Neurodegenerative Diseases. ASN Neuro.

[30] Panfoli I., Puddu A., Bertola N., Ravera S., Maggi D. (2021). The Hormetic Effect of Metformin:“Less Is More”?. Int. J. Mol. Sci..

[31] Yang M., Wei X., Yi X., Jiang D.-S. (2024). Mitophagy-Related Regulated Cell Death: Molecular Mechanisms and Disease Implications. Cell Death Dis..

[32] Di Magno L., Di Pastena F., Bordone R., Coni S., Canettieri G. (2022). The Mechanism of Action of Biguanides: New Answers to a Complex Question. Cancers.

[33] Fontaine E. (2018). Metformin-Induced Mitochondrial Complex I Inhibition: Facts, Uncertainties, and Consequences. Front. Endocrinol..

[34] Vial G., Detaille D., Guigas B. (2019). Role of Mitochondria in the Mechanism(s) of Action of Metformin. Front. Endocrinol..

[35] Crescenzi E., Leonardi A., Pacifico F. (2023). Iron Metabolism in Cancer and Senescence: A Cellular Perspective. Biology.

[36] Santana-Codina N., Mancias J.D. (2018). The Role of NCOA4-Mediated Ferritinophagy in Health and Disease. Pharmaceuticals.

[37] Wu Y., Zhang Z., Ren M., Chen Y., Zhang J., Li J., Gao F., Bao Y., Huang Y., Yang X. (2025). Metformin Induces Apoptosis and Ferroptosis of Ovarian Cancer Cells Under Energy Stress Conditions. Cells.

[38] Fang X., Ardehali H., Min J., Wang F. (2023). The Molecular and Metabolic Landscape of Iron and Ferroptosis in Cardiovascular Disease. Nat. Rev. Cardiol..

[39] Gao M., Monian P., Pan Q., Zhang W., Xiang J., Jiang X. (2016). Ferroptosis Is an Autophagic Cell Death Process. Cell Res..

[40] Doll S., Proneth B., Tyurina Y.Y., Panzilius E., Kobayashi S., Ingold I., Irmler M., Beckers J., Aichler M., Walch A. (2017). ACSL4 Dictates Ferroptosis Sensitivity by Shaping Cellular Lipid Composition. Nat. Chem. Biol..

[41] Liang D., Minikes A.M., Jiang X. (2022). Ferroptosis at the Intersection of Lipid Metabolism and Cellular Signaling. Mol. Cell.

[42] Merkel M., Goebel B., Boll M., Adhikari A., Maurer V., Steinhilber D., Culmsee C. (2023). Mitochondrial Reactive Oxygen Species Formation Determines ACSL4/LPCAT2-Mediated Ferroptosis. Antioxidants.

[43] Bersuker K., Hendricks J.M., Li Z., Magtanong L., Ford B., Tang P.H., Roberts M.A., Tong B., Maimone T.J., Zoncu R. (2019). The CoQ Oxidoreductase FSP1 Acts Parallel to GPX4 to Inhibit Ferroptosis. Nature.

[44] Jiang L., Kon N., Li T., Wang S.-J., Su T., Hibshoosh H., Baer R., Gu W. (2015). Ferroptosis as a P53-Mediated Activity during Tumour Suppression. Nature.

[45] Zhang W., Sun Y., Bai L., Zhi L., Yang Y., Zhao Q., Chen C., Qi Y., Gao W., He W. (2021). RBMS1 Regulates Lung Cancer Ferroptosis through Translational Control of SLC7A11. J. Clin. Investig..

[46] Chen X., Xiao S., Zhang Z., Gong J., Wu J., Wu J., Jin L., Lyu J., Ren X. (2026). Regulation of Mitochondrial Iron Homeostasis in Tumor Cells. Mol. Med..

[47] Wu K., El Zowalaty A.E., Sayin V.I., Papagiannakopoulos T. (2024). The Pleiotropic Functions of Reactive Oxygen Species in Cancer. Nat. Cancer.

[48] Hu Z., Zhao Y., Li L., Jiang J., Li W., Mang Y., Gao Y., Dong Y., Zhu J., Yang C. (2023). Metformin Promotes Ferroptosis and Sensitivity to Sorafenib in Hepatocellular Carcinoma Cells via ATF4/STAT3. Mol. Biol. Rep..

[49] Sternadt D., Pereira-Martins D.A., Chatzikyriakou P., Albuquerque-Simões L., Yang M., Silveira D.R.A., Wierenga A.T.J., Weinhäuser I., Hogeling S.M., Oudejans L.L. (2026). Metformin Induces Ferroptosis Associated with Lipidomic Remodeling in AML. Blood Adv..

[50] Gu D., Sun Y., Wang J., Sun J., Lou H., Kang W. (2025). Metformin Regulates Ferroptosis in Skin Cutaneous Melanoma via ATF3/NRF2 Axis. Cancer Genet..

[51] Viswanathan V.S., Ryan M.J., Dhruv H.D., Gill S., Eichhoff O.M., Seashore-Ludlow B., Kaffenberger S.D., Eaton J.K., Shimada K., Aguirre A.J. (2017). Dependency of a Therapy-Resistant State of Cancer Cells on a Lipid Peroxidase Pathway. Nature.

[52] Lei G., Zhuang L., Gan B. (2024). The Roles of Ferroptosis in Cancer: Tumor Suppression, Tumor Microenvironment, and Therapeutic Interventions. Cancer Cell.

[53] Villalón-García I., Povea-Cabello S., Álvarez-Córdoba M., Talaverón-Rey M., Suárez-Rivero J.M., Suárez-Carrillo A., Munuera-Cabeza M., Reche-López D., Cilleros-Holgado P., Piñero-Pérez R. (2022). Vicious Cycle of Lipid Peroxidation and Iron Accumulation in Neurodegeneration. Neural Regen. Res..

[54] Levi S., Ripamonti M., Moro A.S., Cozzi A. (2024). Iron Imbalance in Neurodegeneration. Mol. Psychiatry.

[55] Koenig A.M., Mechanic-Hamilton D., Xie S.X., Combs M.F., Cappola A.R., Xie L., Detre J.A., Wolk D.A., Arnold S.E. (2017). Effects of the Insulin Sensitizer Metformin in Alzheimer Disease. Alzheimer Dis. Assoc. Disord..

[56] AlRasheed H.A., Bahaa M.M., Elmasry T.A., Elberri E.I., Kotkata F.A., El Sabaa R.M., Elmorsi Y.M., Kamel M.M., Negm W.A., Hamouda A.O. (2025). Randomized, Double-Blind, Placebo-Controlled Pilot Study of Metformin as an Adjunctive Therapy in Parkinson’s Disease. Front. Pharmacol..

[57] De Keersmaecker A.V., Van Doninck E., Popescu V., Willem L., Cambron M., Laureys G., D’ Haeseleer M., Bjerke M., Roelant E., Lemmerling M. (2024). A Metformin Add-on Clinical Study in Multiple Sclerosis to Evaluate Brain Remyelination and Neurodegeneration (MACSiMiSE-BRAIN): Study Protocol for a Multi-Center Randomized Placebo Controlled Clinical Trial. Front. Immunol..

[58] Ng T.P., Feng L., Yap K.B., Lee T.S., Tan C.H., Winblad B. (2014). Long-Term Metformin Usage and Cognitive Function among Older Adults with Diabetes. J. Alzheimer’s Dis..

[59] Orkaby A.R., Cho K., Cormack J., Gagnon D.R., Driver J.A. (2017). Metformin vs Sulfonylurea Use and Risk of Dementia in US Veterans Aged $65 Years with Diabetes. Neurology.

[60] Campbell J.M., Stephenson M.D., De Courten B., Chapman I., Bellman S.M., Aromataris E. (2018). Metformin Use Associated with Reduced Risk of Dementia in Patients with Diabetes: A Systematic Review and Meta-Analysis. J. Alzheimer’s Dis..

[61] Samaras K., Makkar S., Crawford J.D., Kochan N.A., Wen W., Draper B., Trollor J.N., Brodaty H., Sachdev P.S. (2020). Metformin Use Is Associated With Slowed Cognitive Decline and Reduced Incident Dementia in Older Adults With Type 2 Diabetes: The Sydney Memory and Ageing Study. Diabetes Care.

[62] Sluggett J.K., Koponen M., Simon Bell J., Taipale H., Tanskanen A., Tiihonen J., Uusitupa M., Tolppanen A.M., Hartikainen S. (2020). Metformin and Risk of Alzheimer’s Disease among Community-Dwelling People with Diabetes: A National Case-Control Study. J. Clin. Endocrinol. Metab..

[63] Wu L., Zhang Y., Wang G., Ren J. (2024). Molecular Mechanisms and Therapeutic Targeting of Ferroptosis in Doxorubicin-Induced Cardiotoxicity. Basic Transl. Sci..

[64] Wu Z., Bai Y., Chang C., Jiao Y., Chen Q., Guo Z. (2025). Metformin Attenuates Myocardial Ischemia/Reperfusion-Induced Ferroptosis through the Upregulation of Nur77-Mediated IDH1. Biochim. Biophys. Acta (BBA)-Mol. Cell Res..

[65] Tsurusaki S., Tsuchiya Y., Koumura T., Nakasone M., Sakamoto T., Matsuoka M., Imai H., Kok C.Y.-Y., Okochi H., Nakano H. (2019). Hepatic Ferroptosis Plays an Important Role as the Trigger for Initiating Inflammation in Nonalcoholic Steatohepatitis. Cell Death Dis..

[66] Zhao X., Yan L., Tian L., Zhang X., Liu R., Li Z. (2025). Metformin Improves Intestinal Ischemia-Reperfusion Injury by Reducing the Formation of Mitochondrial Associated Endoplasmic Reticulum Membranes (MAMs) and Inhibiting Ferroptosis in Intestinal Cells. Front. Pharmacol..

[67] Kajbaf F., De Broe M.E., Lalau J.-D. (2016). Therapeutic Concentrations of Metformin: A Systematic Review. Clin. Pharmacokinet..

[68] Graham G.G., Punt J., Arora M., Day R.O., Doogue M.P., Duong J., Furlong T.J., Greenfield J.R., Greenup L.C., Kirkpatrick C.M. (2011). Clinical Pharmacokinetics of Metformin. Clin. Pharmacokinet..

[69] Zhou K., Donnelly L.A., Kimber C.H., Donnan P.T., Doney A.S.F., Leese G., Hattersley A.T., McCarthy M.I., Morris A.D., Palmer C.N.A. (2009). Reduced-Function SLC22A1 Polymorphisms Encoding Organic Cation Transporter 1 and Glycemic Response to Metformin: A GoDARTS Study. Diabetes.

[70] Chen L., Shu Y., Liang X., Chen E.C., Yee S.W., Zur A.A., Li S., Xu L., Keshari K.R., Lin M.J. (2014). OCT1 Is a High-Capacity Thiamine Transporter That Regulates Hepatic Steatosis and Is a Target of Metformin. Proc. Natl. Acad. Sci. USA.

[71] Shu Y., Sheardown S.A., Brown C., Owen R.P., Zhang S., Castro R.A., Ianculescu A.G., Yue L., Lo J.C., Burchard E.G. (2007). Effect of Genetic Variation in the Organic Cation Transporter 1 (OCT1) on Metformin Action. J. Clin. Investig..

[72] Li J., Yang Z., Tuo B. (2019). Role of OCT1 in Hepatocellular Carcinoma. Onco. Targets. Ther..

[73] Amengual-Cladera E., Morla-Barcelo P.M., Morán-Costoya A., Sastre-Serra J., Pons D.G., Valle A., Roca P., Nadal-Serrano M. (2024). Metformin: From Diabetes to Cancer—Unveiling Molecular Mechanisms and Therapeutic Strategies. Biology.

[74] Kiełbowski K., Król M., Bakinowska E., Pawlik A. (2024). The Role of ABCB1, ABCG2, and SLC Transporters in Pharmacokinetic Parameters of Selected Drugs and Their Involvement in Drug–Drug Interactions. Membranes.

[75] Chaker F., Kallel A., Khessairi N., Yazidi M., Oueslati I., Chatti H.A., Feki M., Chihaoui M. (2025). Metformin Efficacy and Tolerance According to Genetic Polymorphisms of Organic Cation Transporter 1 in Tunisian Patients with Type 2 Diabetes. Front. Endocrinol..

[76] Schwabedissen H.E., Verstuyft C., Kroemer H.K., Becquemont L., Kim R.B. (2010). Human Multidrug and Toxin Extrusion 1 (MATE1/SLC47A1) Transporter: Functional Characterization, Interaction with OCT2 (SLC22A2), and Single Nucleotide Polymorphisms. Am. J. Physiol. Physiol..

[77] Madiraju A.K., Erion D.M., Rahimi Y., Zhang X.-M., Braddock D.T., Albright R.A., Prigaro B.J., Wood J.L., Bhanot S., MacDonald M.J. (2014). Metformin Suppresses Gluconeogenesis by Inhibiting Mitochondrial Glycerophosphate Dehydrogenase. Nature.

[78] Gormsen L.C., Sundelin E.I., Jensen J.B., Vendelbo M.H., Jakobsen S., Munk O.L., Hougaard Christensen M.M., Brøsen K., Frøkiær J., Jessen N. (2016). In Vivo Imaging of Human {11C}-Metformin in Peripheral Organs: Dosimetry, Biodistribution, and Kinetic Analyses. J. Nucl. Med..

[79] Chandel N.S., Avizonis D., Reczek C.R., Weinberg S.E., Menz S., Neuhaus R., Christian S., Haegebarth A., Algire C., Pollak M. (2016). Are Metformin Doses Used in Murine Cancer Models Clinically Relevant?. Cell Metab..

[80] He L., Wondisford F.E. (2015). Metformin Action: Concentrations Matter. Cell Metab..

[81] Madiraju A.K., Qiu Y., Perry R.J., Rahimi Y., Zhang X.-M., Zhang D., Camporez J.-P.G., Cline G.W., Butrico G.M., Kemp B.E. (2018). Metformin Inhibits Gluconeogenesis via a Redox-Dependent Mechanism in Vivo. Nat. Med..

[82] Hou Y., Cai S., Yu S., Lin H. (2021). Metformin Induces Ferroptosis by Targeting MiR-324-3p/GPX4 Axis in Breast Cancer. Acta Biochim. Biophys. Sin..

[83] Fan R., Deng A., Lin R., Zhang S., Cheng C., Zhuang J., Hai Y., Zhao M., Yang L., Wei G. (2024). A Platinum (IV)–Artesunate Complex Triggers Ferroptosis by Boosting Cytoplasmic and Mitochondrial Lipid Peroxidation to Enhance Tumor Immunotherapy. MedComm.

[84] Dixon S.J., Patel D.N., Welsch M., Skouta R., Lee E.D., Hayano M., Thomas A.G., Gleason C.E., Tatonetti N.P., Slusher B.S. (2014). Pharmacological Inhibition of Cystine–Glutamate Exchange Induces Endoplasmic Reticulum Stress and Ferroptosis. eLife.

[85] Gan B. (2023). How Erastin Assassinates Cells by Ferroptosis Revealed. Protein Cell.

[86] Tang K., Chen Q., Liu Y., Wang L., Lu W. (2022). Combination of Metformin and Sorafenib Induces Ferroptosis of Hepatocellular Carcinoma Through P62-Keap1-Nrf2 Pathway. J. Cancer.

[87] Sun Y., Berleth N., Wu W., Schlutermann D., Deitersen J., Stuhldreier F., Berning L., Friedrich A., Akgun S., Mendiburo M.J. (2021). Fin56-Induced Ferroptosis Is Supported by Autophagy-Mediated GPX4 Degradation and Functions Synergistically with MTOR Inhibition to Kill Bladder Cancer Cells. Cell Death Dis..

[88] Gaschler M.M., Andia A.A., Liu H., Csuka J.M., Hurlocker B., Vaiana C.A., Heindel D.W., Zuckerman D.S., Bos P.H., Reznik E. (2018). FINO2 Initiates Ferroptosis through GPX4 Inactivation and Iron Oxidation. Nat. Chem. Biol..

[89] Zhang B., Yu Z., Zhang J., Xu Y., Zhang M., Dai Z., Zhu J., Zheng S. (2025). Identification of Androgen Receptor as a Molecular Docking Target for Survival and Response to Metformin-Induced Ferroptosis in Liver Cancer. Cancer Rep..

[90] Lang X., Green M.D., Wang W., Yu J., Choi J.E., Jiang L., Liao P., Zhou J., Zhang Q., Dow A. (2019). Radiotherapy and Immunotherapy Promote Tumoral Lipid Oxidation and Ferroptosis via Synergistic Repression of SLC7A11. Cancer Discov..

[91] Tadokoro T., Ikeda M., Ide T., Deguchi H., Ikeda S., Okabe K., Ishikita A., Matsushima S., Koumura T., Yamada K.-I. (2020). Mitochondria-Dependent Ferroptosis Plays a Pivotal Role in Doxorubicin Cardiotoxicity. JCI Insight.

[92] Du Y., Zhu Y.-J., Zhou Y.-X., Ding J., Liu J.-Y. (2022). Metformin in Therapeutic Applications in Human Diseases: Its Mechanism of Action and Clinical Study. Mol. Biomed..

[93] Osorio-Llanes E., Villamizar-Villamizar W., Ospino Guerra M.C., D\’\iaz-Ariza L.A., Castiblanco-Arroyave S.C., Medrano L., Mengual D., Belón R., Castellar-López J., Sepúlveda Y. (2023). Effects of Metformin on Ischemia/Reperfusion Injury: New Evidence and Mechanisms. Pharmaceuticals.

[94] Borra S.S., Jane N.R., Palaniappan D., Subramanian R., Patankar M.A., Krishnamoorthy S.G., Parthasarathy A.K. (2023). Genetic Polymorphism of Organic Cation Transporter 2 (OCT2) and Its Effects on the Pharmacokinetics and Pharmacodynamics of Metformin: A Narrative Review. Egypt. J. Med. Hum. Genet..

[95] Isop L.M., Neculau A.E., Necula R.D., Kakucs C., Moga M.A., Dima L. (2023). Metformin: The Winding Path from Understanding Its Molecular Mechanisms to Proving Therapeutic Benefits in Neurodegenerative Disorders. Pharmaceuticals.

[96] Zake D.M., Kurlovics J., Zaharenko L., Komasilovs V., Klovins J., Stalidzans E. (2021). Physiologically Based Metformin Pharmacokinetics Model of Mice and Scale-up to Humans for the Estimation of Concentrations in Various Tissues. PLoS ONE.

[97] Shen G., Liu J., Wang Y., Deng Z., Deng F. (2025). Ferroptosis in Cancer and Inflammatory Diseases: Mechanisms and Therapeutic Implications. MedComm.

[98] Mishima E., Nakamura T., Doll S., Proneth B., Fedorova M., Pratt D.A., Friedmann Angeli J.P., Dixon S.J., Wahida A., Conrad M. (2025). Recommendations for Robust and Reproducible Research on Ferroptosis. Nat. Rev. Mol. Cell Biol..

[99] Wang Z., Zhang Y., Xu Y., Yang M., Wei Y., Li Y., Yang T., Wang X., Yang C., Feng Q. (2026). Emerging Role of Ferroptosis in Diabetes and Associated Complications: When Metabolic Dysregulation Meets Cell Death. Cell Prolif..

